# Perceiving societal pressure to be happy is linked to poor well-being, especially in happy nations

**DOI:** 10.1038/s41598-021-04262-z

**Published:** 2022-02-17

**Authors:** Egon Dejonckheere, Joshua J. Rhee, Peter K. Baguma, Oumar Barry, Maja Becker, Michał Bilewicz, Thomas Castelain, Giulio Costantini, Girts Dimdins, Agustín Espinosa, Gillian Finchilescu, Malte Friese, Maria Cecilia Gastardo-Conaco, Angel Gómez, Roberto González, Nobuhiko Goto, Peter Halama, Camilo Hurtado-Parrado, Gabriela M. Jiga-Boy, Johannes A. Karl, Lindsay Novak, Liisi Ausmees, Steve Loughnan, Khairul A. Mastor, Neil McLatchie, Ike E. Onyishi, Muhammad Rizwan, Mark Schaller, Eleonora Serafimovska, Eunkook M. Suh, William B. Swann, Eddie M. W. Tong, Ana Torres, Rhiannon N. Turner, Alexander Vinogradov, Zhechen Wang, Victoria Wai-lan Yeung, Catherine E. Amiot, Watcharaporn Boonyasiriwat, Müjde Peker, Paul A. M. Van Lange, Christin-Melanie Vauclair, Peter Kuppens, Brock Bastian

**Affiliations:** 1 Department of Medical and Clinical Psychology, Tilburg University, Warandelaan 2, 5037 AB Tilburg, The Netherlands; 2 Faculty of Psychology and Educational Sciences, KU Leuven, Tiensestraat 102, Leuven, Belgium; 3 Melbourne School of Psychological Sciences, The University of Melbourne, Melbourne, Australia; 4 School of Psychology, Makerere University, Kampala, Uganda; 5 Department of Psychology, University Cheikh Anta Diop, Dakar, Senegal; 6 CLLE, Université de Toulouse, CNRS, Toulouse, France; 7 Faculty of Psychology, University of Warsaw, Warszawa, Poland; 8 Instituto de Investigaciones Psicológicas, Universidad de Costa Rica, San Pedro Montes de Oca, Costa Rica; 9 Department of Psychology, University of Milan-Bicocca, Milan, Italy; 10 Department of Psychology, University of Latvia, Riga, Latvia; 11 Department of Psychology, Pontificia Universidad Católica del Perú, Lima, Peru; 12 Department of Psychology, University of the Witwatersrand, Johannesburg, South Africa; 13 Department of Psychology, Saarland University, Saarbrücken, Germany; 14 Department of Psychology, University of the Philippines, Diliman, Philippines; 15 Department of Social and Organizational Psychology, Universidad Nacional de Educación a Distancia, Madrid, Spain; 16 Escuela de Psicología, Pontificia Universidad Católica de Chile, Santiago, Chile; 17 Department of Psychology, Kyoto Notre Dame University, Kyoto, Japan; 18 Center of Social and Psychological Sciences, Slovak Academy of Sciences, Bratislava, Slovakia; 19 Department of Psychology, Konrad Lorenz University and Troy University, Bogotá, Colombia; 20 School of Psychology, Swansea University, Swansea, UK; 21 School of Psychology, Victoria University of Wellington, Wellington, New Zealand; 22 Department of Psychology, University of Illinois, Chicago, USA; 23 Institute of Psychology, University of Tartu, Tartu, Estonia; 24 Department of Psychology, University of Edinburgh, Edinburgh, UK; 25 School of Liberal Studies, Universiti Kebangsaan Malaysia, Bangi, Malaysia; 26 Psychology Department, Lancaster University, Lancaster, UK; 27 Department of Psychology, University of Nigeria, Nsukka, Nigeria; 28 Department of Psychology, University of Haripur, Haripur, KPK Pakistan; 29 Department of Psychology, University of British Columbia, Vancouver, Canada; 30 Institute for Sociological, Political and Juridical Research, University of Ss Cyril and Methodius, Skopje, North Macedonia; 31 Department of Psychology, Yonsei University, Seoul, South Korea; 32 Department of Psychology, University of Texas, Austin, USA; 33 Department of Psychology, National University of Singapore, Singapore, Singapore; 34 Department of Psychology, Federal University of Paraíba, Paraíba, Brazil; 35 School of Psychology, Queen’s University Belfast, Belfast, UK; 36 Department of Psychology, Taras Shevchenko National University of Kyiv, Kyiv, Ukraine; 37 School of Social Development and Public Policy, Fudan University, Shanghai, China; 38 Department of Applied Psychology, Lingnan University, Tuen Mun, Hong Kong; 39 Department of Psychology, Université du Québec À Montréal, Montreal, Canada; 40 Faculty of Psychology, Chulalongkorn University, Bangkok, Thailand; 41 Department of Psychology, MEF University, Istanbul, Turkey; 42 Department of Experimental and Applied Psychology, Vrije Universiteit Amsterdam, Amsterdam, The Netherlands; 43 Centre for Psychological Research and Social Intervention, Instituto Universitário de Lisboa (ISCTE-IUL), CIS-IUL, Lisboa, Portugal

**Keywords:** Human behaviour, Depression

## Abstract

Happiness is a valuable experience, and societies want their citizens to be happy. Although this societal commitment seems laudable, overly emphasizing positivity (versus negativity) may create an unattainable emotion norm that ironically compromises individual well-being. In this multi-national study (40 countries; 7443 participants), we investigate how societal pressure to be happy and not sad predicts emotional, cognitive and clinical indicators of well-being around the world, and examine how these relations differ as a function of countries’ national happiness levels (collected from the World Happiness Report). Although detrimental well-being associations manifest for an average country, the strength of these relations varies across countries. People’s felt societal pressure to be happy and not sad is particularly linked to poor well-being in countries with a higher World Happiness Index. Although the cross-sectional nature of our work prohibits causal conclusions, our findings highlight the correlational link between social emotion valuation and individual well-being, and suggest that high national happiness levels may have downsides for some.

Humans value happiness. Around the world, individuals share a similar aspiration to lead a satisfying and happy life^1^, yet there is also an emerging recognition that this personal quest in itself may have well-being consequences. Placing a premium on the value of positive emotion is known to paradoxically undermine our well-being, not only as a function of how we value happiness ourselves^2–4^, but also as a function of how the society we live in emphasizes the importance of being happy^5–7^.

Preliminary work suggests that the deleterious effects of pursuing happiness may vary across nations^8,9^. Here, we aim to provide a robust cross-national test of this (predictive) effect, investigating whether the experienced social value placed on happiness ironically relates to poorer well-being across a large sample of nations. We also examine a previously unexplored source of variance between countries, their global levels of self-reported happiness, assessed with an established metric for societal well-being, the World Happiness Index (WHI^10^). By examining the link between social valuation of emotion and well-being across the globe, we aim to provide further insight into the important link between culture and individual emotional functioning^11,12^.

## The societal pressure to be happy and subjective well-being

Happiness or in scientific terms, high subjective well-being^13^ is advantageous and desired, not in the least because it signals accomplishment and optimal functioning^14^. For individuals, high subjective well-being is associated with personal thriving in various life domains (e.g., work, social relations, physical health^15–17^). But also, for nations more broadly, social indicators research consistently illustrates that happy inhabitants indicate societal flourishing on economic, social, and political fronts^18^. Together, these favorable outcomes explain people’s natural tendency to value happiness, both for themselves and their fellow man.

Although this social engagement with happiness appears admirable, recent research also highlights the risks of overly promoting positive emotion, which can result in a felt social pressure to be happy^19–23^. Today, the message that happiness is an important life goal is expressed at many different levels in modern societies, and social-emotion research shows that people readily internalize these salient emotion standards^24–26^. On a macro-level, for example, the prominence of happiness is evidenced explicitly by the numerous happiness coaches, campaigns and self-help books that provide us with tips and tricks to cultivate the most positive mindset^27^, but also more implicitly, by the seemingly perfect lives of influencers on social media^28^, and the ubiquity of smiling faces and happiness allusions in prime-time commercials and magazines^29,30^. On a micro-level, people may feel pressured by their friends, family or colleagues to present themselves in an overly positive way, because these close social contacts directly or indirectly encourage them to feel happy^31,32^. At last, this subjective experience may even exist in the absence of concrete, objective antecedents^33^. Regardless of the specific mechanism, this one-sided social emphasis on happiness also risks simultaneously cultivating the perception that there is little room for negativity^9^. Indeed, in many modern societies or social groups, the natural experience of negative emotion is easily stigmatized^34^, regarded as maladaptive for our mental well-being^35^, and as something troublesome that instantly needs cure^36,37^. Also in this case, social others may shape the internal expectation that negativity is undesired^24,25^. Nevertheless, occasional feelings of stress, sadness or anxiety are an inevitable reality for every human being, making it virtually impossible to constantly comply with the apparent stringent norm to be happy^5^. Because this unattainable standard readily reveals discrepancies between our actual emotional life and the emotions society apparently approves of, the perceived failure to meet social expectations is known to trigger negative meta-emotions, pessimistic self-attitudes and ruminative responding^7,20,38,39^, with the resulting ironic aggravation of these undesired emotional states^40,41^.

Eventually, the chronic failure to adhere to these unrealistic emotion standards may compromise people’s well-being, as demonstrated by a large body of correlational and experimental research with various indicators of subjective well-being^13^. Emotionally, the experimental induction to value happiness (e.g., via happiness-extolling mock articles or verbal communication) paradoxically elicits blunted positive emotional responding to enjoyable events^3^, increased rumination over negative emotion^7^, and stronger feelings of loneliness^22^. On the flip side, experiencing societal pressure to avoid negativity (e.g., induced via mock articles that emphasize the social cost of negative emotion) instigates increased negative emotion (both in terms of intensity and duration^9^) and equally triggers loneliness^19^. Cognitively, the societal valuation of positive emotions (and the perceived devaluation of negative ones) relates to lower life satisfaction judgments for people who occasionally feel negative^9,21^. Finally, in the clinical realm, excessively valuing positivity has been linked to more depressive symptoms in both adolescent^23^ and adult samples^42^, and compared to healthy controls, depressed patients hold stronger beliefs that they should feel more positive and less negative^43^. Within individuals, perceiving social pressure not to feel negative paradoxically predicts increases in depressive symptomatology over time^5^.

An important question that currently remains unanswered is to what extent the detrimental link between the felt social pressure to be happy and individual well-being is universal versus culture-specific^11,12^. The vast majority of cited studies are typically confined to single-nation (Western) samples (e.g., ^5,19,20^). In the few cases where cultural variation is central to their investigation, researchers mainly relied on a small sample of different geographical regions (e.g., United States, Germany, Russia, East Asia^8^) or their study was limited to different nationalities living in the same country (e.g., Australian and East Asian students living in Australia^9^). Therefore, a comprehensive and cross-national evaluation of the tendency to place a social premium on happiness and the associated well-being problems with doing so, together with the examination of potential country-level moderators, is a crucial next step in understanding the link between social emotion valuation and individual adaptive functioning^21^.

## Country-level happiness: The World Happiness Index

Although there are many avenues through which the social value placed on happiness may be inadvertently communicated and reinforced, it is possible that the happiness seen in other members of society may aggravate the ironic and negative (predictive) well-being effects of the felt social pressure to achieve personal happiness^44–46^. Signs of human happiness can manifest in a multitude of ways^47^, and are not limited to the explicit expression of overt joyful behavior alone (e.g., smiling facial expressions, positive verbal communication, etc.). Happiness is also evident in other more subtle, implicit overt cues (e.g., having more social contact, engaging in pleasurable activities, etc.), and finally also includes truly covert experiences of joy and related behaviors (e.g., *feeling* happy, providing a high happiness rating in a well-being survey, etc.).

If the happiness that is displayed by other citizens adds to the personal pressure to be happy or amplifies its (predictive) well-being effects, then national levels of self-reported happiness within a given society could pick up on this process. One of the most prominent and established barometers to evaluate national levels of self-reported happiness is the WHI, an annual metric published by the Sustainable Development Solution Network commissioned by the United Nations^10^. Based on the subjective happiness ratings of a large-scale and nationally representative sample (collected by the Gallup World Poll^48^), this initiative aims to present a global ranking of the most happy and unhappy nations in the world.

At its core, the WHI is thought to summarize how happy the average person within a country typically feels^10^. However, it is equally possible that in countries in which citizens report higher levels of happiness, people, on average, also experience more social pressure to be happy and not sad, because social norms prescribing the value of happiness are elevated within these countries. If this hypothesis is correct, we should expect a meaningful country-level relation between the average perceived social pressure to be happy within a country and its national WHI score.

Second, the possibility exists that in countries with higher national happiness levels, people’s own personal failure to (at times) live up to society’s prescribed standard to be happy may be accentuated by other people’s actual happiness. Based on an integration of the previously cited body of socio-cultural^6–8^ and (meta-)emotional^38–43^ research, it is possible that, for some individuals, the happiness seen in others may set up a forced social comparison context^44–46^ in which discrepancies between one’s own emotional life and society’s perceived expectations are more painfully apparent, because others seemingly comply with the prevailing standard to be happy with little trouble. In this regard, social network research shows that happiness is distributed unequally within societies^49,50^, and this imbalance in happiness could create the detrimental basis for social comparison in a population. Indeed, for people who regularly experience negative emotion, being confronted with happy people inevitably highlights the fact that their feelings are out of step with the emotional lives of others^46^, and this self-other incongruity could aggravate the negative (predictive) well-being effects of the felt social pressure to strive for happiness^7,20,45^. If this rationale is correct, we should expect that the negative relation between people’s perceived social pressure to pursue happiness and their well-being is ironically stronger in high WHI countries.

## The current study

To determine how the perceived social pressure to pursue happiness relates to people’s subjective well-being around the world, we conducted a large-scale cross-national study (40 countries; 7,443 participants). In a first step, we examined whether the detrimental well-being associations of this felt pressure replicated across a wide array of countries. We surveyed for both participants’ perceived social pressure to be happy^51^, as well as not to be depressed or anxious^20^. Regarding their subjective well-being, we acknowledged the multi-componential structure of this construct^13,52^. In line with established conventions on how to survey subjective well-being^53^, we considered both emotional (i.e., the frequency and intensity of positive [PA] and negative affect [NA]), cognitive (i.e., life satisfaction) and clinical indicators (i.e., depressive, anxiety-, and stress-related symptomatology).

In a second step, we examined the role of nations’ global happiness levels as a potential source of between-country variance explaining the negative well-being associations of the felt social pressure to feel positive and not negative. To this end, we obtained a global WHI score for each participating country from the World Happiness Report^10^. This score is based on the average life evaluation of a nationally representative sample^48^, using the Cantril Ladder^54^. Respondents are asked to evaluate the quality of their current life on a 11-rung ladder that ranges from worst possible life (zero) to best possible life (ten). Consequently, the WHI is more an indication of the average life satisfaction displayed by the inhabitants of a particular country, rather than their global subjective well-being^10^.

First, we explored whether higher national WHI scores are associated with stronger felt social pressure to be happy and not anxious or depressed. Second, we examined the moderating role of countries’ WHI score on the relation between this felt social pressure and people’s well-being. We hypothesized that the perceived societal pressure to feel happy and not anxious or depressed ironically shows stronger detrimental relations with people’s subjective well-being in high WHI nations. In these happy contexts, the painful observation that other people are (seemingly) emotionally able to live up to society’s expectations when you yourself are unable to do so, likely makes personal deviations from the desired emotion standard more salient^24,44^. In this sense, the negative (predictive) well-being effects of the felt social pressure to strive for happiness (and avoid sadness) are likely to be reinforced in high WHI countries.

To evaluate how the perceived societal pressure to feel (a) positive and (b) not negative is linked to poor subjective well-being, we performed two (separate) series of multilevel models (participants nested within countries) with the different subjective well-being indicators as outcomes of interest. Although previous work established a causal and unidirectional effect of experiencing pressure to be happy and not sad on subjective well-being (e.g., ^7,9^), we acknowledge that our selection of outcomes and predictors in the current cross-sectional multilevel context is somewhat arbitrary, and that we are ultimately restricted to correlational claims (but see *SI* 6, where we show that this arbitrary decision does not impact our conclusions). For each model, we examined the random effect distributions of these types of pressure in the prediction of well-being to see if detrimental links manifest globally or whether nation-specific relations appear. Next, to explore the role of countries’ WHI score, we examined its country-level relation with the average felt social pressure within a country, and evaluated its cross-level interaction with people’s felt social pressure to see whether national happiness levels moderated the within-country relations with well-being (see Methods for more detailed information about our statistical analyses).

## Results

### Descriptive statistics

Before answering the research questions central to this investigation, different elements in Table 1 deserve special consideration. First, within nations, the interrelation between the social pressure to feel positive and not negative is moderately positive (*r* = 0.54, *p* < 0.001). This suggests a common factor in the perceived social pressure to pursue positivity and to avoid negativity, but also underscores the uniqueness of both constructs. Second, in line with previous research^2–6,9,19,21,23^, both types of social pressure show the expected pattern of associations with all well-being indicators. Feeling social pressure to be happy and not sad is associated with reduced life satisfaction, experiencing less frequent and intense positive, but more frequent and intense negative emotions, and more symptoms of depression, anxiety and stress (|*r*|s ≥ 0.05, *ps* ≤ 0.050). Finally, between countries, national WHI scores are only significantly related to countries’ average life satisfaction levels (*r* = 0.37, *p* = 0.017); but not with emotional or clinical markers of well-being. This confirms the convergent (and discriminant) validity of this country-level metric, because the WHI only assesses countries’ average life satisfaction and not global subjective well-being.

**Table 1:** Summary statistics and correlations among all measures.

Variables	Descriptive statistics				Correlations										
	Mean (SD)	ICC	α _within_	α _between_	1	2	3	4	5	6	7	8	9	10	WHI
**Cognitive subjective well-being**															
1. Life satisfaction	4.30 (1.29)	.06	.80	.89		.19	− .05	.33 *	.04	− .12	− .10	.16	.04	.30	.37 *
**Emotional subjective well-being**															
2. PA Frequency	5.60 (1.54)	.03	.74	.83	.47 ***		− .35 *	.85 ***	− .51 ***	− .37 *	− .09	− .42 **	− .05	− .27	− .21
3. NA Frequency	4.74 (1.85)	.08	.75	.92	− .38 ***	− .43 ***		− .29	.93 ***	.64 ***	.58 ***	.64 ***	.54***	.44 **	.23
4. PA Intensity	5.60 (1.66)	.03	.77	.83	.39 ***	.76 ***	− .35 ***		− .32 *	− .40 *	− .14	− .35 *	.05	− .02	− .21
5. NA intensity	4.80 (1.96)	.06	.77	.89	− .34 ***	− .41 ***	.82 ***	− .22 ***		.57 ***	.49 **	.63 ***	.48**	.48 **	.29
**Clinical subjective well-being**															
6. Depression	1.75 (0.65)	.12	.83	.95	− .44 ***	− .48 ***	.63 ***	− .39 ***	.58 ***		.90***	.88 ***	.22	.26	.06
7. Anxiety	1.69 (0.60)	.11	.78	.95	− .23 ***	− .33 ***	.55 ***	− .25 ***	.52 ***	.63 ***		.79 ***	.32 *	.21	− .06
8. Stress	2.00 (0.61)	.12	.80	.95	− .25 ***	− .40 ***	.61 ***	− .31 ***	.58 ***	.65 ***	.70 ***		.23	.41 **	.23
**Perceived emotion norm**															
9. SEHS	6.01 (1.34)	.11	.75	.94	− .05 *	− .10 ***	.27 ***	− .08 **	.24 ***	.21 ***	.22 ***	.24 ***		.63 ***	.23
10. SEDAS	5.56 (1.18)	.11	.72	.95	− .21 ***	− .23 ***	.31 ***	− .18 ***	.29 ***	.27 ***	.23 ***	.26 ***	.54 ***		.26

ICC = Intra-class correlation, ratio of between-country variance to total variance. Within- and between-country multilevel internal consistencies (α) were calculated following^79^. Correlations below the diagonal represent the average within-country correlation between people’s personal scores, correlations above the diagonal represent the between-country correlations between country means (i.e., national scores). WHI = World Happiness Index; PA = Positive Affect; NA = Negative Affect; SEHS = Social Expectancies to be Happy Scale; SEDAS = Social Expectancies not to feel Depressed or Anxious Scale; *p ≤ .05, **p ≤ .01, ***p ≤ .001.

### Universal versus nation-specific subjective well-being effects

The fixed effects in Table 2 indicate how the perceived social pressure to be happy and not anxious or depressed (separately) relate to all subjective well-being markers for the average country in our sample. These results are fully in line with the average within-nation correlations in Table 1. Within the average country, the social pressure to be happy and not sad is linked to lower life satisfaction judgements (*β*s ≤ −0.05, *p*s ≤ 0.024). Emotionally, experiencing these types of social pressure relate to less frequent and intense positive, but more frequent and intense negative emotions (|*β*|s ≥ 0.09, *p*s ≤ 0.003, R%s = 100%). Finally, in the clinical realm, feeling pressured to be happy and not sad predicts stronger symptoms of depression, anxiety and general distress (*β*s ≥ 0.09, *p*s < 0.001). The (predictive) well-being effects of the felt social pressure not to feel negative are typically stronger than to feel positive.

**Table 2:** Exploring the universality of the detrimental well-being effects of the perceived social pressure to be happy and not to be depressed or anxious.

	Perceived social pressure to be happy					Perceived social pressure not to be depressed or anxious				
	Fixed effect	SD random effects	# Positive	# Negative	# Null	Fixed effect	SD random effects	# Positive	# Negative	# Null
**Cognitive subjective well-being**										
Life satisfaction	− 0.05*	0.11	5	**10**	25	− 0.23***	0.10	5	**24**	10
**Emotional subjective well-being**										
PA Frequency	− 0.11***	0.14	4	**14**	22	− 0.27***	0.15	13	**18**	8
NA Frequency	0.36***	0.09	**36**	0	4	0.46***	0.08	**35**	0	4
PA Intensity	− 0.09**	0.13	3	**10**	27	− 0.24***	0.13	3	**28**	8
NA intensity	0.36***	0.10	**32**	0	8	0.46***	0.09	**34**	0	5
**Clinical subjective well-being**										
Depression	0.10***	0.05	**32**	0	8	0.14***	0.04	**33**	0	6
Anxiety	0.09***	0.03	**33**	1	6	0.11***	0.03	**31**	0	8
Stress	0.10***	0.04	**34**	0	6	0.12***	0.04	**32**	1	6

Each fixed effect represents the observed relation for the average country in our sample. The standard deviation of the random effects distribution describes the observed variability around that average association. For each well-being variable, we report the number of significant positive, significant negative and null-associations across countries (*n* = 40 for the perceived social pressure to be happy; *n* = 39 for the perceived social pressure not to be depressed or anxious, due to an irreversible coding error for Poland). Both types of pressure were within-country centered. The number of associations that mirror the fixed effect are bolded. PA = Positive Affect; NA = Negative Affect; *p ≤ .05, **p ≤ .01, ***p ≤ .001.

However, when examining the variability in random effects, we observe considerable between-country heterogeneity for some well-being indicators. This points towards a potential moderating impact of critical nation-level variables that exacerbate or alleviate the debilitating link between the felt social pressure to be happy and not sad and personal well-being. In particular, country-specific patterns appear for positive markers of subjective well-being (i.e., life satisfaction, PA frequency and intensity). For these indicators, the average negative relation with both types of social pressure significantly switches sign in 3 to 13 of the countries in our sample (and is non-significant in 10 to 28 countries). In contrast, for negative markers of subjective well-being (i.e., clinical symptoms, NA frequency and intensity), the average positive association with these types of pressure only turns negative in maximum 1 country (and is non-significant in 4 to 8 countries), suggesting a more universal detrimental association.

### The moderating role of national happiness levels

To explain this between-country variability in (predictive) well-being effects, we examine the role of nations’ global levels of self-reported happiness on the perceived social pressure to be happy and not sad in two ways. First, inspecting the correlations above the diagonal in Table 1, national WHI scores are not significantly related to the average felt societal pressure within a country (*r*s ≤ 0.23, *p*s ≥ 0.112). Thus, contrary to what we predicted, the perceived societal norms that prescribe people to feel happy and not anxious or depressed are not particularly elevated in countries with a high WHI score.

**Figure 1:**
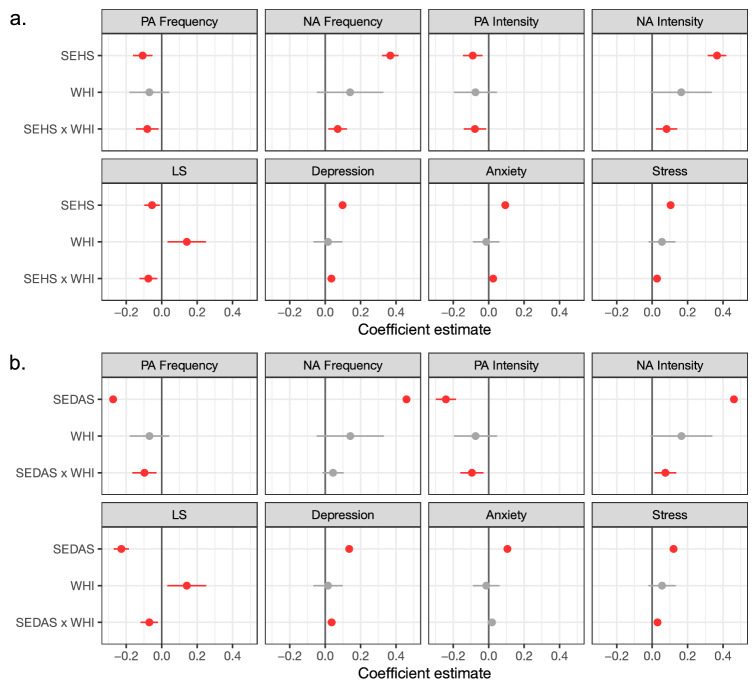
Exploring the differential role of the perceived social pressure (**a**) to be happy and (**b**) not to be depressed or anxious in various well-being indicators as function of countries’ World Happiness Index (WHI). Dots represent the magnitude of the fixed effects in each multilevel model (red = significant; gray = non-significant with α = .05), error bars refer to the 95% confidence interval. Intercepts are not presented for optimal visibility, but were always significant with *p*s ≤ .001. Original values, standard errors, test statistics and *p*-values can be found in Supplementary Tables 2 and 3. PA = Positive Affect; NA = Negative Affect; LS = Life Satisfaction; SEHS = Social Expectancies to be Happy Scale; SEDAS = Social Expectancies not to feel Depressed or Anxious Scale.

Second, however, when exploring the moderating impact of national happiness levels, we observe how the within-country association between almost all subjective well-being indicators and the felt societal pressure to be happy and not sad changes as a function of a country’s WHI score (see Fig. 1). For the perceived social pressure to be happy, significant cross-level interactions with nations’ WHI score indicate that these effects are stronger in countries that report higher levels of national happiness. In line with our hypothesis, perceiving social pressure to be happy is linked to poorer subjective well-being in high WHI countries, both emotionally (|*β*|s ≥ 0.07, *p*s ≤ 0.016, R%s ≤ 87%), cognitively (*β* = −0.08, *p* = 0.003) and clinically (*β*s ≥ 0.03, *p*s ≤ 0.006).

**Figure 2:**
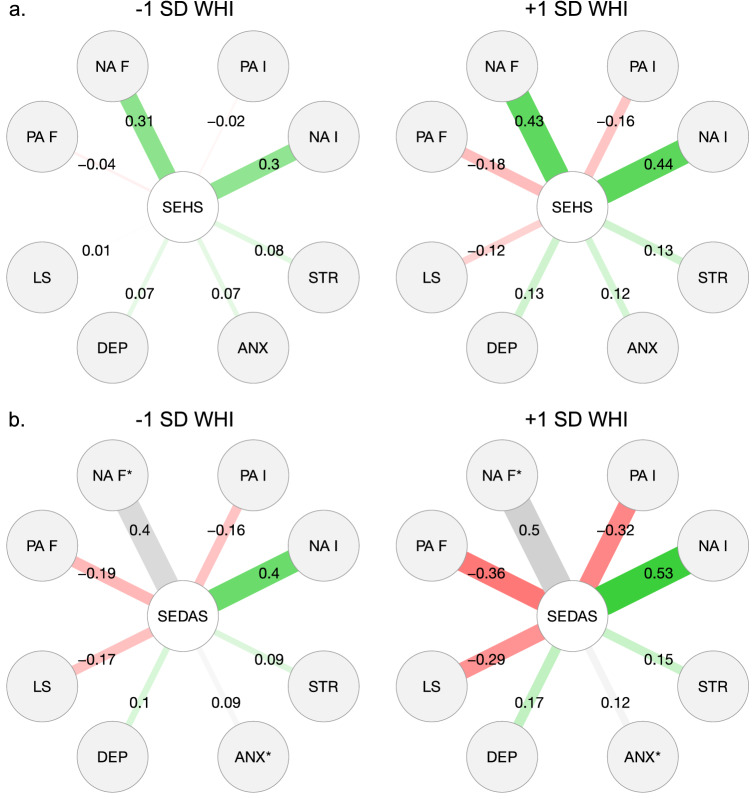
The predictive effect of people’s perceived social pressure (**A**) to be happy and (**B**) not to be depressed or anxious for all well-being indicators in high and low WHI countries (− 1/ + 1 SD). The magnitude and transparency of the edges corresponds with the strength of the association. Green lines represent positive relations, red lines negative relations. Gray lines indicate that the cross-level interaction was non-significant, meaning that the person-level relation between the perceived emotion norm and subjective well-being did not meaningfully differ in low versus high WHI countries (also denoted with an *). WHI = World Happiness Index; SEHS = Social Expectancies to be Happy Scale; SEDAS = Social Expectancies not to feel Depressed or Anxious Scale; PA = Positive Affect; NA = Negative Affect; F = Frequency; I = Intensity; LS = Life Satisfaction; DEP = Depressive symptoms, ANX = Anxiety symptoms, STR = Stress symptoms.

Indeed, as can be seen from Panel A in Fig. 2, comparing the countries in our sample with a lower (− 1 *SD*) versus higher (+ 1 *SD*) WHI score, the link between people’s perceived social pressure to be happy and their subjective well-being is substantially stronger in the latter. In terms of absolute magnitude differences, the absolute explanatory effect of the perceived social pressure to be happy in people’s well-being is almost always small to non-existent in low WHI nations (|*β*|s ≤ 0.08; except for the prediction of the frequency and intensity of NA feelings). In contrast, in high WHI nations, the absolute predictive effect of this pressure ranges from 0.12 to 0.44. In terms of relative magnitude, the (absolute) difference in predictive effects between low and high WHI countries is the smallest for the clinical indicators (difference in |*β*|s ≤ 0.06), and the largest for the emotional indicators of psychological well-being (difference in |*β*|s ≥ 0.12).

**Figure 3:**
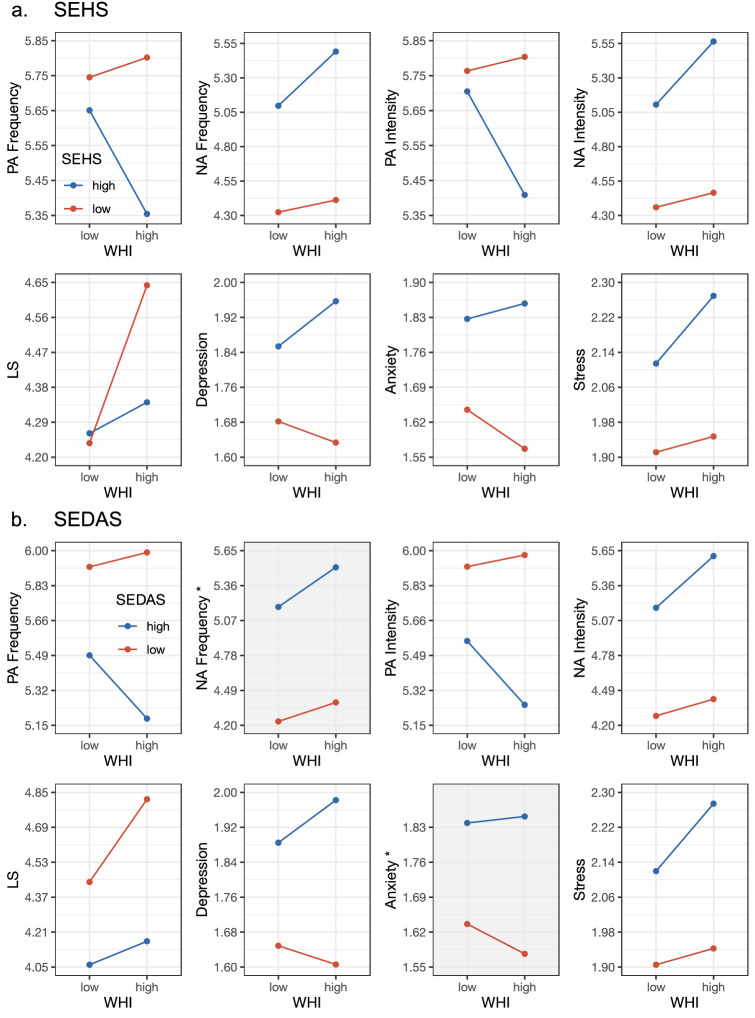
Unfolding all cross-level interactions between countries’ WHI score and participants’ perceived social pressure (**a**) to be happy and (**b**) not to be depressed or anxious in the prediction of individual subjective well-being. To distinguish between low (− 1 SD) and high (+ 1 SD) values, we adopted the average within-country SD for person-level predictors, and the between-country SD for countries’ WHI score. Gray plots indicate that the cross-level interaction was not significant (also denoted with an *). WHI = World Happiness Index; SEHS = Social Expectancies to be Happy Scale; SEDAS = Social Expectancies not to feel Depressed or Anxious Scale; PA = Positive Affect; NA = Negative Affect; LS = Life Satisfaction.

Finally, the graphical visualization of these significant cross-level interactions in Fig. 3 (Panel A) further unfolds the moderating impact of national WHI scores. Independent of WHI status, the perceived social pressure to be happy predicts poorer subjective well-being in all indicators. However, in high WHI countries this prediction is always stronger, generally producing larger differences in well-being in happier nations between people who experience little versus a great deal of social pressure to be happy.

For the felt societal pressure not to be depressed or anxious, we observe a pattern of results that is highly similar. Here, significant cross-level interactions with countries’ WHI score indicate that most negative (predictive) well-being effects of participants’ perceived societal pressure to not feel negative are stronger in countries that report higher levels of national happiness. Emotionally, in countries with a high WHI score, the perceived societal pressure to avoid negative emotion shows stronger ties to a reduced experience of PA (both in terms of frequency and intensity; *β*s ≤ −0.09, *p*s ≤ 0.005, R%s ≤ 93%) and an increased experience of NA (in terms of intensity, *β* = 0.07, *p* = 0.017, R% = 67%; but not frequency, *β* = 0.04, *p* = 0.146, R% = 80%). Cognitively, this societal pressure predicts poor life satisfaction, particularly in happy nations (*β* = −0.07, *p* = 0.005). Clinically, in high WHI countries, feeling socially pressured not to feel depressed or anxious paradoxically predicts more symptoms of depression and general distress (*β*s ≥ 0.03, *p*s ≤ 0.003), but not anxiety (*β* = 0.02, *p* = 0.057).

An explicit comparison of lower (− 1 *SD*) versus higher (+ 1 *SD*) WHI nations in Fig. 2 (Panel B) further elucidates the differences in the strength of these within-country associations. For all well-being indicators, the link with people’s perceived social pressure not to feel anxious or depressed is substantially weaker in low WHI countries, except for the frequency of NA and anxiety symptoms. Regarding absolute magnitude differences, the (absolute) explanatory effect of the perceived social pressure not to feel negative in people’s well-being never exceeds 0.19 in countries with a lower WHI score, except for the frequency and intensity of NA. In contrast, in high WHI countries, the absolute significant predictive effect of this pressure is almost always higher, ranging from 0.15 to 0.53. In terms of relative magnitude, the (absolute) difference in explanatory effects between low and high WHI nations is smallest for anxiety symptoms (difference in *β* = 0.03) and strongest for the frequency in PA (difference in *β* = 0.17).

Finally, Panel B in Fig. 3 again illustrates that, for all well-being indicators, people who experience social pressure not to feel negative always report poorer subjective well-being, irrespective of their country’s WHI score. However, in high WHI nations this link is usually stronger (except for NA frequency and anxiety symptoms), which leads differences in personal well-being in happier countries to be more extreme when comparing citizens who perceive considerable versus little social pressure not to feel negative.

## Discussion

The present cross-national study set out to explore how the perceived social emotion norms to pursue positivity and avert negativity play a role in people’s subjective well-being around the world. Breaking down well-being into its different constituents^13,52^, we robustly demonstrated how the perceived societal premium on happiness (and aversion of sadness) in most countries paradoxically relates to fewer and less intense experiences of positive emotions (with an opposite pattern for negative emotions), lower life satisfaction evaluations, and more symptomatic complaints related to depression, anxiety and general distress.

However, we also demonstrated that these negative (predictive) well-being effects are not entirely universal, corroborating the preliminary findings of earlier studies^8,9^. Particularly for positive markers of subjective well-being, we observed how the (predictive) effects of the felt social pressure to be happy and not sad are subject to substantial national differences. That is, in a great number of countries, feeling socially pressured to be happy and not sad was actually unrelated to positive well-being, and in a small minority an opposite association even emerged. Here, the perceived social premium on happiness was related to higher life satisfaction evaluations, and a more frequent and intense experience of positive emotion. In contrast, for negative indicators of subjective well-being, the (predictive) effects of the felt social pressure to be happy and not sad were more universally negative. In almost all countries, experiencing pressure to be happy and not sad was related to more and stronger negative feelings, and stronger symptoms of depression, anxiety and stress. This difference suggests that the presence of negative well-being (e.g., frequent and intense negative emotions or psychopathological symptoms) likely discloses discrepancies with the prevailing societal emotion standard that are more salient, compared to the absence of positive well-being (e.g., little to low positive emotions or poor life satisfaction). This finding coincides with the observation that experiencing social pressure to avoid negativity overall typically shows poorer well-being associations than the pressure to pursue positivity.

Exploring the factors that drive this country-level variability in (predictive) well-being effects, we found that feeling pressured to be happy and not sad is particularly associated with poor well-being in countries with high national happiness levels. Although these perceived happiness norms in themselves are, on average, not more elevated among citizens of high-ranked WHI nations, the personal belief that social others view the experience of positive emotions as a key indicator of success in life (and devalue negative emotions) is found to hold an especially negative relation to people’s well-being in happier countries.

Regarding actual (causal) process explanations, the cross-sectional nature of our data prevents us to uncover the exact explanatory mechanisms underlying the current moderation, but the idea that the happiness seen in others in high WHI countries could ironically amplify the negative relation between the perceived social pressure to be happy and personal well-being opens up multiple avenues for future research. For example, it is possible that citizens of high WHI nations are typically more expressive of their happiness, as previous cross-cultural studies established national differences in emotional display rules^55^. Not only would this explain a higher WHI ranking for these countries, more overt signs of happiness would also produce a stronger detrimental basis for negative social comparison in unhappy people^7,46^). Second, it could be that high WHI countries also suffer from more happiness inequality^49,50^. Nation-level aggregates do not reveal how happiness is distributed within a country^53^, but a stronger imbalance in happiness would explain why unhappy individuals feel compelled to align their feelings with those of the majority group. If true, this perceived social pressure would not only further impair these outcasts’ personal well-being, it would also again lead to more happiness inequality, ultimately contributing to a self-sustaining feedback mechanism.

Regardless of the specific mechanism, our findings emphasize that an exclusive focus or overreliance on national aggregates may be misleading to inform well-being policy, a concern that has been repeatedly expressed by the social indicator movement in the past^53,56^. Although the WHI is meaningfully related to countries’ average life satisfaction levels, underscoring the construct validity of this index on a national level (i.e., this metric does not echo country-level averages in emotional or clinical well-being^10^), the present results suggest that the WHI may be less equipped to provide insight into the subjective well-being of specific citizens. Indeed, we found that a higher WHI ranking does not necessarily indicate higher subjective well-being for everyone within that country, as the normative emotion processes in high WHI societies may paradoxically work against individual well-being for some. Overall, these findings further illustrate the concern that considerable between-person heterogeneity may undermine the unifying quality of country-level metrics^56,57^, and highlight the importance of additionally considering the within-nation processes that may explain this variability.

Finally, with respect to the potential societal implications of our findings, nationwide (psycho-)educational campaigns that put the pressing need to be happy in perspective, while also acknowledging the valuable role of negative emotion (particularly in high WHI nations), could have beneficial effects for people’s psychological well-being in the long run^2,5^. In this way, the outdated yet dominant societal discourse that promotes a one-sided embrace of one’s emotions can make way for an updated version, in which people learn to appreciate the full scope of their emotional lives, both positive and negative.

### Limitations

The current findings should be considered in the light of some limitations. First, in addition to the fact that our claims about the specific process mechanisms underlying our results remain speculative on the basis of correlational data alone, we acknowledge that country-level WHI scores may not provide the optimal window of analysis to fittingly establish negative social comparison. Although the WHI clearly captures elements of this referential process at a macro-level, future studies with a more fine-grained resolution are needed to complement the current work with findings from micro-level contexts (i.e., social comparison of happiness as a result of immediate social interactions). Previous work on the prevalence of suicide in happy places has shown that both perspectives do not always converge in their results^58,59^, and other paradoxical patterns described in the happiness literature (e.g., the Easterlin Paradox^60^) have highlighted the critical importance of explicitly clarifying the level of analysis when interpreting results. In this regard, future studies could also benefit from explicitly distinguishing between different potential sources that shape people’s perceived social pressure to be happy and not sad (e.g., macro- versus micro-level, implicit versus explicit, objective antecedents versus subjective appraisals, etc.). Although this pressure is likely multi-determined, the instruments currently available do not differentiate between these different factors.

Second, to assess the average life satisfaction in our own sample, we did not include the original WHI Cantril Ladder^10,54^, but rather relied on Diener’s traditional Satisfaction With Life rating scale^61^. Although both instruments are known to correlate highly^62^, we cannot simply generalize our findings to other types of well-being assessments. Similarly, compared to the diverse and nationally representative samples in WHI research^10^, this multi-national study mainly comprised a student population, limiting the generalizability of the found associations to other sub-samples within a country. For example, with respect to different age groups, the factors that contribute to a happy life are known to change remarkably across the life span^63^, and adolescent students are generally more susceptible to the expectations of social others or the influence of peers compared to adults (e.g.,^64,65^). Surveying a more balanced research sample and exploring the moderating role of other theoretically relevant demographic covariates (both on an individual and societal level) will further elucidate the comprehensiveness of the established well-being associations.

Finally, as is the case in all cross-national studies, expecting full language and translation equivalence across countries is difficult (e.g., some emotion words may be interpreted slightly different around the world^66^). Nevertheless, national inequivalence would likely introduce more measurement noise to the data, acting against establishing meaningful associations.

## Materials and methods

### Participants

The present research project was part of a larger cross-national study investigating how individual and cultural values influence emotional well-being and moral attitudes around the world. The initiating sites were based in Australia and Belgium, and the associated researchers contacted potential collaborators via e-mail, in which they outlined the aims and nature of the study, and provided an initial copy of the survey materials (in English). Upon agreeing to participate, all collaborating sites arranged the requisite ethical approval for data collection at their host institution, and translated the questionnaires into their native language (see *SI 1* for more information about this process). The original study was approved by the Psychological Sciences Human Ethics Advisory Group in Australia (1647465.2) and the Social-Societal Ethical Committee KU Leuven in Belgium (G-2017 10 954). Each collaborating site was asked to enroll a minimum sample of 100 university students that originated from the nation of testing (e.g., no international or exchange students).

In the end, we collected data from 40 different countries (42 sites), adequately covering all populated continents in the world (i.e., Europe *n* = 17; Asia *n* = 10; Africa *n* = 4; South America *n* = 4; North America *n* = 3; Oceania *n* = 2). A world map with all participating countries can be found in *SI 2*, together with the final sample size for each site. On average, each country collected 186 participants (*SD* = 129), with a total sample of 7,443 participants taking part in the study (*M*_age_ = 21.81, *SD*_age_ = 5.60). The balance of gender identification consisted of 32% men, 61.2% women, 0.3% other, and 6.5% unspecified, and the majority of participants (87.6%) were enrolled in a psychology course at the time of the study. All participants provided informed consent.

### Procedure and materials

In each country, we adopted a standardized survey battery that was locally translated into participants’ native language (and back-translated by some but not all host institutions; see *SI 1* for more information) to evaluate their subjective well-being, alongside their perception of the predominant emotion norms in their country. Participants were only sampled a single time. Next, accessing the public data of the 2019 World Happiness Report, we obtained a global WHI score for each participating country^10^. Summary statistics and correlations among all measures can be found in Table 1.

#### Emotional well-being components: natural positive and negative affect

To evaluate the emotional components in subjective well-being, the distinct and global experience of positive (PA) and negative affect (NA^67^), we compiled a list of four positive (*happy*, *joyful*, *relaxed*, *calm*) and four negative (*sad*, *depressed*, *stressed*, *anxious*) emotion items, respectively. The selection of these emotions was based on the circumplex model of affect^68^ to ensure an adequate representation of different arousal levels. We invited participants to rate their everyday emotional experience both in terms of frequency (*How often have you experienced the following emotion during the last month?*) and intensity (*How intense was your experience of the following emotion?*), as both dimensions are known to relate differently to subjective well-being^69^. For each emotion item, participants provided their response on a 9-point Likert scale that ranged from *none of the time* (one) to *all of the time* (nine) for frequency, and from *very mild* (one) to *very intense* (nine) for intensity. We averaged same-valenced emotion ratings for each dimension to create a score for PA and NA frequency, and PA and NA intensity.

#### Cognitive well-being component: satisfaction with life

We assessed life satisfaction with the Satisfaction with Life scale^61^. This 5-item questionnaire is designed to capture a broad and integrative evaluation of people’s life (e.g., *The conditions of my life are excellent.*), and concerns the cognitive-judgmental component in subjective well-being^13^. Participants rated each item on a 7-point Likert scale, ranging from *strongly disagree* (one) to *strongly agree* (seven), and we averaged across items to get a global life satisfaction score.

#### Clinical well-being components: mood complaints

To determine the presence of mood-related symptomatology, experiential factors that usually undermine high subjective well-being^52^, participants had to complete the Depression Anxiety and Stress Scale^70^. This 21-item survey is based on the tripartite model of anxiety and depression^71^, and consists of three 7-item subscales that aim to differentiate between prototypical symptoms of depression (e.g., *I felt down-hearted and blue.*), anxiety (e.g., *I felt scared without any good reason.*) and general distress (e.g., *I tended to over-react to situations.*^70^). Participants indicated how frequently they experienced each item over the last week on a 4-point scale that ranged from *not at all* (zero) to *most of the time* (three), and we averaged responses per subscale to get an indication of each symptom type severity.

#### Perceived emotion norms

We assessed participants’ perceived societal expectancies to feel positive with the Social Expectancies about Happiness (SEHS^51^), and not to feel negative with the Social Expectancies about Depression and Anxiety Scale (SEDAS^20^). The SEHS is a 9-item survey that evaluates people’s global idea about how they think their society expects people to pursue positivity (e.g., *I think that society places a great deal of pressure on people to feel happy.* or *People in my society view people who feel happy as more valuable.*; see *SI 3* for the full item list). Conversely, the SEDAS is a 13-item instrument that reveals people’s general beliefs about how they think their society disapproves of negative emotional states such as depression or anxiety (e.g., *I think society tends to place a lot of pressure on people not to feel depressed or anxious.* or *I think society accepts people who feel depressed or anxious as normal.* [reversed]). For both scales, participants rated each statement on a 9-point Likert scale that ranged from *strongly disagree* (one) to *strongly agree* (nine). We averaged across all items (after rescoring the reversed items), so that higher SEHS and SEDAS scores indicated stronger individual beliefs that society pressures people to be happy, and disapproves of negative emotion, respectively. Due to an irreversible coding error the SEDAS scores for Poland are missing.

#### World happiness index

To get a robust indication of the country-level happiness reported within a particular society, we evaluated countries’ WHI score. A country’s WHI score is based on the average life evaluation of a nationally representative sample^10^, using the Cantril Ladder^54^. In this single-item survey, respondents are asked to evaluate the quality of their current life on a 11-rung ladder that ranges from *worst possible life* (zero) to *best possible life* (ten). As such, the WHI is more an indication of the average life satisfaction displayed by the inhabitants of a particular country, rather than their global subjective well-being^10^. Cantril Ladder evaluations and traditional self-report measures for life satisfaction (e.g.,^61^) are known to correlate very high^62^.

Because data collection took place in 2019, we adopted the WHI scores for that year (freely accessible online: https://worldhappiness.report/ed/2019/). The countries that took part in our study representatively covered the global ranking (*M* = 6.11; *SD* = 0.86), with the Netherlands being the highest ranked country in our sample (7.49; position 5) and Uganda the lowest (4.19; position 136 out of 156). For the participating sites in England, Scotland, Wales and Northern Ireland, we imputed the WHI score of the United Kingdom. In all analyses, we used countries’ actual WHI score, not their corresponding ranking.

### Statistical analyses

All analyses in this article were conducted in R (version 4.0.0^72^). To reproduce our results and figures, researchers can consult the data, code and materials at the Open Science Framework (https://osf.io/3aut4/). All methods were carried out in accordance with relevant guidelines and regulations.

#### Multilevel analysis

To account for the hierarchical structure of the data, we performed our analyses in a multilevel framework, using the *lme4* R-package^73^. Specifically, we ran various two-level models, with persons (*n* = 7,443) nested within countries (*n* = 40). In all models, slopes and intercept were allowed to vary randomly across countries to account for possible national differences in the found effects. For an intuitive interpretation of the model parameter estimates, we group-mean centered all person-level predictors. Country-level WHI scores were grand-mean centered. In this way, we effectively separated within- and between-country effects^74^. All statistical tests were two-sided.

To evaluate how the perception of the societal emotion standard in a country differently relates to subjective well-being as a function of nation’s global happiness level, we ran a series of multilevel models with the various well-being indicators as the outcome of interest (i.e., cognitive, emotional and clinical well-being markers). At the person-level, we either entered participants’ perceived societal pressure to feel positive (SEHS) or not to feel negative (SEDAS) as the focal predictor (separately). At the country-level, we introduced the national WHI scores and evaluated the cross-level interactions with the global intercept and person-level predictor. A generic overview of all model formulae can be found in *SI 4*.

We emphasize that our multilevel approach inevitably introduces an asymmetry in the specified relation between outcome and predictor^75^. Because the selection of an outcome and predictor is always somewhat arbitrary with cross-sectional data, we additionally ran all reversed models, together with a third statistical approach in which all variables were within-country standardized (to remove the asymmetry in a multilevel context^76^). Results can be found in *SI 6* and illustrate that this arbitrary decision did not impact our conclusions.

#### Robustness analysis

With respect to the emotional well-being components, we acknowledge that every item operationalization of a PA and NA composite score is somewhat arbitrary. Because there is little theoretical consensus on how researchers should exactly construct these affective aggregates^77^, we performed a leave-one-out multiverse analysis for our PA and NA constructs (e.g.,^78^). For each of the multilevel models that involved PA or NA frequency or intensity as a predictor, we evaluated the robustness of each model parameter under different PA and NA operationalizations. Because we evaluated four specific emotion items for each affective construct, this yielded 15 alternative PA and NA operationalizations, each based on a unique combination of emotion items. We entered each unique affective aggregate as a predictor in the previously outlined models, and evaluated the proportion of models for which the significance test of each estimate (with α = 0.05) yielded identical conclusions as the model in which the PA and NA composites were based on all emotion items (of which the results are presented here). A higher robustness percentage (R%) indicates that the model parameter is less driven by particular PA and NA operationalizations.

## Supplementary Information

Supplementary Information.

## Data Availability

To reproduce our results and figures, researchers can consult all data and materials at the Open Science Framework (https://osf.io/3aut4/).

## Code availability

All analyses in this article were conducted in R (version 4.0.0). To reproduce our results and figures, researchers can consult the code at the Open Science Framework (https://osf.io/3aut4/).

## References

[1] Oishi S, Diener E, Lucas RE (2016). The optimum level of well-being: Can people be too happy?. Perspect. Psychol. Sci..

[2] Ford BQ, Mauss IB (2014). The paradoxical effects of pursuing positive emotion: When and why wanting to feel happy backfires.

[3] Mauss IB, Tamir M, Anderson CL, Savino NS (2011). Can seeking happiness make people happy? Paradoxical effects of valuing happiness. Emotion.

[4] Gruber J, Mauss IB, Tamir M (2011). A dark side of happiness? How, when, and why happiness is not always good. Perspect. Psychol. Sci..

[5] Dejonckheere E, Bastian B, Fried EI, Murphy SC, Kuppens P (2017). Perceiving social pressure not to feel negative predicts depressive symptoms in daily life. Depress. Anxiety.

[6] Bastian B (2013). Normative influences on secondary disturbance: The role of social expectancies. Aust. Psychol..

[7] McGuirk L, Kuppens P, Kingston R, Bastian B (2018). Does a culture of happiness increase rumination over failure?. Emotion.

[8] Ford BQ (2015). Culture shapes whether the pursuit of happiness predicts higher or lower well-being. J. Exp. Psychol. Gen..

[9] Bastian B (2012). Feeling bad about being sad: The role of social expectancies in amplifying negative mood. Emotion.

[10] J. Helliwell, R. Layard, J. Sachs, *World Happiness Report 2019* (Sustainable Development Solutions Network, 2019).

[11] De Vaus J, Hornsey MJ, Kuppens P, Bastian B (2018). Exploring the East-West divide in prevalence of affective disorder: a case for cultural differences in coping with negative emotion. Pers. Soc. Psychol. Rev..

[12] Curhan KB (2014). Just how bad negative affect is for your health depends on culture. Psychol. Sci..

[13] Diener E (1984). Subjective well-being. Psychol. Bull..

[14] Fredrickson BL (2004). The broaden-and-build theory of positive emotions. Philos. Trans. R. Soc. Lond. B Biol. Sci..

[15] Fisher CD (2010). Happiness at Work. Int. J. Manag. Rev..

[16] Ramsey MA, Gentzler AL (2015). An upward spiral: Bidirectional associations between positive affect and positive aspects of close relationships across the life span. Dev. Rev..

[17] Steptoe A (2019). Happiness and health. Annu. Rev. Public Health.

[18] Veenhoven R (1988). The utility of happiness. Soc. Indic. Res..

[19] Bastian B (2015). Sad and alone: Social expectancies for experiencing negative emotions are linked to feelings of loneliness. Soc. Psychol. Personal. Sci..

[20] Dejonckheere E, Bastian B (2020). Perceiving social pressure not to feel negative is linked to a more negative self-concept. J. Happiness Stud..

[21] Bastian B, Kuppens P, De Roover K, Diener E (2014). Is valuing positive emotion associated with life satisfaction?. Emotion.

[22] Mauss IB (2012). The pursuit of happiness can be lonely. Emotion.

[23] Gentzler AL, Palmer CA, Ford BQ, Moran KM, Mauss IB (2019). Valuing happiness in youth: associations with depressive symptoms and well-being. J. Appl. Dev. Psychol..

[24] Carver CS, Scheier MF (1990). Origins and functions of positive and negative affect: a control-process view. Psychol. Rev..

[25] Rojas M, Veenhoven R (2013). Contentment and affect in the estimation of happiness. Soc. Indic. Res..

[26] Tsai JL (2007). Ideal affect: cultural causes and behavioral consequences. Perspect. Psychol. Sci..

[27] M. E. P. Seligman, *Authentic happiness: Using the new positive psychology to realize your potential for lasting fulfillment*, Reprint edition (Atria Books, 2004).

[28] de Lenne O, Vandenbosch L, Eggermont S, Karsay K, Trekels J (2020). Picture-perfect lives on social media: A cross-national study on the role of media ideals in adolescent well-being. Media Psychol..

[29] Dzuhrina I (2020). Music component on TVCs: Coca Cola “Open Happiness” campaign. Medio.

[30] Mogilner C, Aaker J, Kamvar SD (2012). How happiness affects choice. J. Consum. Res..

[31] Abraham-Smith K, Keville S (2015). The influence of women’s perceived entitlement to have postnatal depression on the disclosure process. Br. J. Midwifery..

[32] Linzbach L, Suojanen I (2020). Behind the happiness mask.

[33] Jussim L (1991). Social perception and social reality: A reflection-construction model. Psychol. Rev..

[34] Wahl OF (1999). Mental health consumers’ experience of stigma. Schizophr. Bull..

[35] Horwitz AV, Wakefield JC (2007). The loss of sadness: How psychiatry transformed normal sorrow into depressive disorder.

[36] Conrad P (2003). Medicalization and social control. Annu. Rev. Sociol..

[37] Touburg G, Veenhoven R (2015). Mental health care and average happiness: Strong effect in developed nations. Adm. Policy Ment. Health..

[38] Bailen NH, Wu H, Thompson RJ (2019). Meta-emotions in daily life: Associations with emotional awareness and depression. Emotion.

[39] Pool GJ, Wood WM, Leck KM (1998). The self-esteem motive in social influence: agreement with valued majorities and disagreement with derogated minorities. J. Pers. Soc. Psychol..

[40] Moberly NJ, Watkins ER (2008). Ruminative self-focus and negative affect: an experience sampling study. J. Abnorm. Psychol..

[41] Nolen-Hoeksema S (1991). Responses to depression and their effects on the duration of depressive episodes. J. Abnorm. Psychol..

[42] Ford BQ, Shallcross AJ, Mauss IB, Floerke VA, Gruber J (2014). Desperately seeking happiness: Valuing happiness is associated with symptoms and diagnosis of depression. J. Soc. Clin. Psychol..

[43] Thompson RJ, Kircanski K, Gotlib IH (2016). The grass is not as green as you think: affect evaluation in people with internalizing disorders. J. Affect. Disord..

[44] A. Tesser, “Emotion in social comparison and reflection processes” in *Social comparison: Contemporary theory and research*, (Lawrence Erlbaum Associates, Inc, 1991), pp. 115–145.

[45] E. Diener, F. Fujita, “Social comparisons and subjective well-being” in *Health, coping, and well-Being: Perspectives from social comparison theory*, (Lawrence Erlbaum Associates Publishers, 1997), pp. 329–357.

[46] Swallow SR, Kuiper NA (1988). Social comparison and negative self-evaluations: An application to depression. Clin. Psychol. Rev..

[47] Diener E, Ryan K (2009). Subjective well-being: a general overview. S. Afr. J. Psychol..

[48] R. D. Tortora, R. Srinivasan, N. Esipova, “The Gallup world poll” in *Survey methods in multinational, multiregional, and multicultural contexts*, (John Wiley & Sons, Ltd, 2010), pp. 535–543.

[49] J. H. Fowler, N. A. Christakis, Dynamic spread of happiness in a large social network: Longitudinal analysis over 20 years in the Framingham Heart Study, *BMJ,* **337**, a2338 (2008). 10.1136/bmj.a2338 PMC2600606 19056788

[50] Veenhoven R (2005). Inequality of happiness in nations. J. Happiness Stud..

[51] B. Bastian, E. Dejonckheere, P. Kuppens, The social pressure to be happy scale: A validation study (in preparation).

[52] Headey B, Kelley J, Wearing A (1993). Dimensions of mental health: Life satisfaction, positive affect, anxiety and depression. Soc. Indic. Res..

[53] J. E. Stiglitz, A. Sen, J. P. Fitoussi, Report by the commission on the measurement of economic performance and social progress. (2009).

[54] H. Cantril, *The pattern of human concerns*, First edition (Rutgers University Press, 1965).

[55] Matsumoto D, Yoo SH, Fontaine J (2008). Mapping expressive differences around the world: The relation between emotional display rules and individualism versus collectivism. J. Cross. Cult..

[56] Chakraborty A (2002). Issues in social indicators, composite indices and inequality. Econ. Polit. Wkly..

[57] Fisher AJ, Medaglia JD, Jeronimus BF (2018). Lack of group-to-individual generalizability is a threat to human subjects research. Proc. Natl. Acad. Sci. U S A.

[58] Daly M, Oswald A, Wilson D, Wu S (2011). Dark contrasts: The paradox of high rates of suicide in happy places. J. Econ. Behav. Organ..

[59] Pendergast PM, Wadsworth T, Kubrin CE (2019). Suicide in happy places: Is there really a paradox?. J. Happiness Stud..

[60] R. Easterlin, Does economic growth improve the human lot? in *Nations an households in economic growth: Essays in honour of Moses Abramovitz,* (New York Academic).

[61] Diener E, Emmons RA, Larsen RJ, Griffin S (1985). The satisfaction with life scale. J. Pers. Assess..

[62] Organisation for economic co-operation and development (OECD), *OECD Guidelines on measuring subjective well-being* (OECD Publishing, 2013). 24600748

[63] Bardo AR (2017). A life course model for a domains-of-life approach to happiness: evidence from the United States. Adv. Life Course Res..

[64] Gardner M, Steinberg L (2005). Peer influence on risk taking, risk preference, and risky decision making in adolescence and adulthood: an experimental study. Dev. Psychol..

[65] S. Ciranka, W. vandenbos. Social influence in adolescent decision-making: A formal framework. *Front. Psychol.* **10,** 1915 (2019). 10.3389/fpsyg.2019.01915 PMC6727856 31555164

[66] Mesquita B, Frijda NH (1992). Cultural variations in emotions: a review. Psychol. Bull..

[67] Pavot W, Diener E (1993). The affective and cognitive context of self-reported measures of subjective well-being. Soc. Indic. Res..

[68] Russell JA (1980). A circumplex model of affect. J. Pers. Soc. Psychol..

[69] E. Diener, E. Sandvik, W. Pavot, “Happiness is the frequency, not the intensity, of positive versus negative affect” in *Assessing well-being: The collected works of ed diener*, Soc. Indic. Res series, (Springer Science & Business Media, 2009), pp. 213–231.

[70] S. H. Lovibond, P. F. Lovibond, *Manual for the depression anxiety stress scales* (The Psychology Foundation of Australia, 1995).

[71] Clark LA, Watson D (1991). Tripartite model of anxiety and depression: psychometric evidence and taxonomic implications. J. Abnorm. Psychol..

[72] RStudio Team, *RStudio: Integrated development for R.* (PBC, 2020).

[73] D. Bates, M. Mächler, B. Bolker, S. Walker, Fitting linear mixed-effects models using **Lme4**. *J. Stat. Soft.* **67** (2015).

[74] S. W. Raudenbush, A. S. Bryk, *Hierarchical linear models: Applications and data analysis methods*, 2nd edition (Sage Publications, Inc, 2001).

[75] Ong AD, Zautra AJ, Finan PH (2017). Inter- and intra-individual variation in emotional complexity: Methodological considerations and theoretical implications. Curr. Opin. Behav. Sci..

[76] Schuurman NK, Ferrer E, de Boer-Sonnenschein M, Hamaker EL (2016). How to compare cross-lagged associations in a multilevel autoregressive model. Psychol. Methods.

[77] E. Dejonckheere, *et al.*, Complex affect dynamic measures add limited information to the prediction of psychological well-being. *Nat. Hum. Bevah.* (2019). 10.1038/s41562-019-0555-0 30988484

[78] Dejonckheere E (2018). The bipolarity of affect and depressive symptoms. J. Pers. Soc. Psychol..

[79] Nezlek JB (2017). A practical guide to understanding reliability in studies of within-person variability. J. Res. Person..

