# Supplementary material for: Perceiving societal pressure to be happy is linked to poor well-being, especially in happy nations
Source: Sci Rep. 2022 Feb 17;12:1514. doi: 10.1038/s41598-021-04262-z (PMC8854619; doi:10.1038/s41598-021-04262-z)
Supplement: Supplementary file 1 — Supplementary Information. [file 41598_2021_4262_MOESM1_ESM.docx]

**Perceiving societal pressure to be happy is linked to poor well-being, especially in happy nations**

**-**

**Supplementary Information**

Egon Dejonckheere^1^, Joshua Rhee^2^, Peter K. Baguma^3^, Oumar Barry^4^, Maja Becker^5^, Michał Bilewicz^6^, Thomas Castelain^7^, Giulio Costantini^8^, Girts Dimdins^9^, Agustín Espinosa^10^, Gillian Finchilescu^11^, Malte Friese^12^, Maria Cecilia Gastardo-Conaco^13^, Angel Gómez^14^, Roberto González^15^, Nobuhiko Goto^16^, Peter Halama^17^, Camilo Hurtado-Parrado^18^, Gabriela M. Jiga-Boy^19^, Johannes A. Karl^20^, Lindsay Novak^21^, Liisi Ausmees^22^, Steve Loughnan^23^, Khairul A. Mastor^24^, Neil McLatchie^25^, Ike E. Onyishi^26^, Muhammad Rizwan^27^, Mark Schaller^28^, Eleonora Serafimovska^29^, Eunkook M. Suh^30^, William B. Swann, Jr^31^, Eddie M. W. Tong^32^, Ana Torres^33^, Rhiannon N. Turner^34^, Alexander Vinogradov^35^, Zhechen Wang^36^, Victoria Wai-lan Yeung^37^, Catherine E. Amiot^38^, Watcharaporn Boonyasiriwat^39^, Müjde Peker^40^, Paul A. M. Van Lange^41^, Christin-Melanie Vauclair^42^, Peter Kuppens^1^, & Brock Bastian^2^

^1^ KU Leuven – Faculty of Psychology and Educational Sciences

^2^ The University of Melbourne – Melbourne School of Psychological Sciences

^3^ Makerere University – School of Psychology

^4^ University Cheikh Anta Diop, Dakar, Senegal – Department of Psychology

^5^ CLLE, Université de Toulouse – CNRS, FR.

^6^ University of Warsaw – Faculty of Psychology

^7^ University of Costa Rica – Instituto de Investigaciones Psicológicas.

^8^ University of Milan-Bicocca – Department of Psychology

^9^ University of Latvia – Department of Psychology

^10^ Pontificia Universidad Católica del Perú – Department of Psychology

^11^ University of the Witwatersrand, South Africa – Department of Psychology

^12^ Saarland University – Department of Psychology

^13^ University of the Philippines, Diliman – Department of Psychology

^14^ Universidad Nacional de Educación a Distancia – Department of Social and Organizational Psychology

^15^ Pontificia Universidad Católica de Chile – Escuela de Psicología, Mide UC

^16^ Kyoto Notre Dame University – Department of Psychology

^17^ Slovak Academy of Sciences – Center of Social and Psychological Sciences

^18^ Konrad Lorenz University and Troy University – Department of Psychology

^19^ Swansea University – Department of Psychology

^20^ Victoria University of Wellington – School of Psychology

^21^ University of Illinois at Chicago – Department of Psychology

^22^ University of Tartu – Institute of Psychology

^23^ University of Edinburgh – Department of Psychology

^24^ Universiti Kebangsaan Malaysia – Center for Liberal Studies

^25^ Lancaster University – Psychology Department

^26^ University of Nigeria, Nsukka – Department of Psychology

^27^ The Delve Pvt Ltd – Department of Research and Assessment

^28^ University of British Columbia – Department of Psychology

^29^ University of Ss Cyril and Methodius, Skopje – Institute for Sociological, Political and Juridical Research

^30^ Yonsei University – Department of Psychology

^31^ University of Texas at Austin – Department of Psychology

^32^ National University of Singapore – Department of Psychology

^33^ Federal University of Paraíba, Brazil – Department of Psychology

^34^ Queen’s University Belfast – School of Psychology

^35^ Taras Shevchenko National University of Kyiv – Department of Psychology

^36^ Fudan University – School of Social Development and Public Policy

^37^ Lingnan University – Department of Applied Psychology

^38^ Université du Québec à Montréal – Department of Psychology

^39^ Chulalongkorn University – Faculty of Psychology

^40^ MEF University, Istanbul – Department of Psychology

^41^ Vrije Universiteit Amsterdam – Department of Experimental and Applied Psychology

^42^ Instituto Universitário de Lisboa (ISCTE-IUL), CIS-IUL, Lisboa, Portugal – Centre for Psychological Research and Social Intervention

Correspondence concerning these supplementary materials should be addressed to Egon Dejonckheere, Faculty of Psychology and Educational Sciences, KU Leuven, Tiensestraat 102, Leuven, 3000, Belgium. E-mail: egon.dejonckheere@kuleuven.be. Supplemental data and code for this article can be accessed here: https://osf.io/3aut4/.

**Supplementary Information**

Supplemental Materials 1 – Translation and back-translation procedure 05

Supplemental Materials 2 – World map and table with participating countries 06

Supplemental Materials 3 – The Social Expectancies to be Happy Scale (SEHS) 08

Supplemental Materials 4 – Generic model structure 09

Supplemental Materials 5 – Summary of all multilevel coefficients 10

Supplemental Materials 6 – Statistical alternatives to evaluate robustness of found effects 12

**Supplemental Materials 1: Translation and back-translation procedure**

With the exception of Italy, Senegal, and nations where the project leaders collected data themselves (i.e., Australia and Belgium), all countries participated in a standardized translation and back-translation protocol for the translation of the survey materials into participants’ native language. First, the collaborators at the relevant site arranged a translation of the original English questionnaires, followed by an independent English back-translation of the translated survey also arranged by the local collaborators. It was emphasized to give priority to loyalty of meaning and familiarity of the content instead of loyalty to the original language (i.e., a decentering approach to translate cross-cultural materials; 59). Next, the project leaders in Australia reviewed this back-translation for any deviations in meaning from the original English survey. In line with the decentering approach, they did not require a strict (back-)translation, rather they preferred surveys that preserved the original meaning of the instrument. All suggested changes were marked on the back-translation and sent back to the relevant collaborators for review. Upon receiving the back-translation, the collaborators provided responses to the project leaders’ comments, including whether the identified issues did indeed derivate from the original meaning, or whether they were simply an artefact of the back-translation process. Finally, these responses were again reviewed by the project leaders in Australia, before final clearance was given to the collaborators to begin local data collection with the translated survey materials.

**Supplemental Materials 2: World map and table with participating countries**


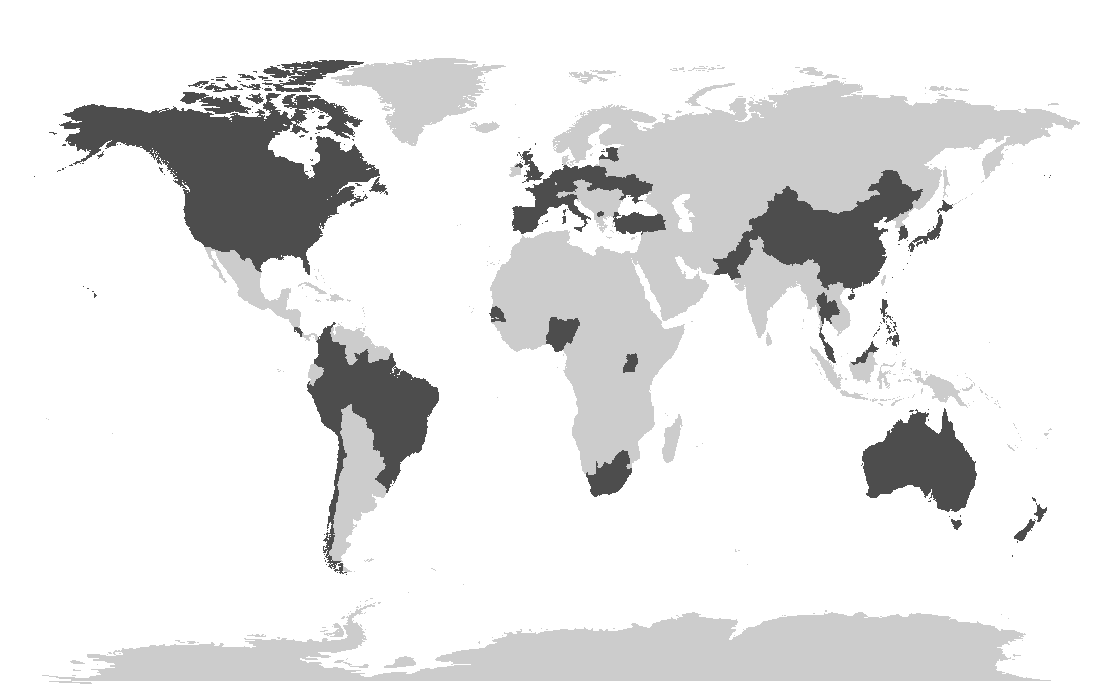


**Supplemental Figure 1.** A world map of all participating countries (*n* = 40). In alphabetic order: Australia, Belgium, Brazil, Canada, Chile, China, Colombia, Costa Rica, England, Estonia, France, Germany, Hong Kong, Italy, Japan, Latvia, Macedonia, Malaysia, The Netherlands, New-Zealand, Nigeria, Northern Ireland, Pakistan, Peru, The Philippines, Poland, Portugal, Scotland, Senegal, Singapore, Slovakia, South-Africa, South-Korea, Spain, Thailand, Turkey, Uganda, Ukraine, United States of America, Wales. This world map was created with the ‘ggplot2’ package in the free and open-source R platform (version 4.0.0; 72).

| **Supplemental Table 1.** Participating countries per continent (*n* = 40). | | |
| --- | --- | --- |
| Country | Sample size | Language |
| **Europe** | | |
| Belgium | 225 | Dutch |
| England | 149 | English |
| Estonia | 63 | Estonian |
| France | 191 | French |
| Germany | 143 | German |
| Italy | 222 | Italian |
| Latvia | 120 | Latvian |
| Macedonia | 124 | Macedonian |
| Netherlands | 110 | Dutch |
| Northern Ireland | 110 | English |
| Poland | 116 | Polish |
| Portugal | 137 | Portuguese |
| Scotland | 110 | English |
| Slovakia | 115 | Slovak |
| Spain | 156 | Spanish |
| Ukraine | 110 | Ukrainian |
| Wales | 85 | English |
| **Asia** | | |
| China | 235 | Chinese |
| Hong Kong | 211 | English |
| Japan | 182 | Japanese |
| Malaysia | 175 | Malay |
| Pakistan | 147 | English |
| Philippines | 151 | English |
| Singapore | 102 | English |
| South Korea | 127 | Korean |
| Thailand | 99 | Thai |
| Turkey | 275 | Turkish |
| **Africa** | | |
| Nigeria | 233 | English |
| Senegal | 778 | French |
| South Africa | 423 | English |
| Uganda | 101 | English |
| **South America** | | |
| Brazil | 138 | Portuguese |
| Chile | 69 | Spanish |
| Colombia | 151 | Spanish |
| Peru | 200 | Spanish |
| **North America** | |  |
| Canada * | 318 | English and French |
| Costa Rica | 130 | Spanish |
| United States of America * | 392 | English |
| **Oceania** |  |  |
| Australia | 386 | English |
| New-Zealand | 134 | English |

**Note.** Countries denoted with an * indicate that two sites collected data for the study.

**Supplemental Materials 3: The Social Expectancies to be Happy Scale (SEHS)**

| **Strongly Disagree**  **1** | **2** | **Moderately Disagree**  **3** | **4** | **Neither Agree or Disagree**  **5** | **6** | **Moderately Agree**  **7** | **8** | **Strongly**  **Agree**  **9** |
| --- | --- | --- | --- | --- | --- | --- | --- | --- |

| 1. Most people think that feeling **Happy** is a key indicator of success in life. | 1 | 2 | 3 | 4 | 5 | 6 | 7 | 8 | 9 |
| --- | --- | --- | --- | --- | --- | --- | --- | --- | --- |
| 2. People in my society view people who feel **Happy** as more valuable. | 1 | 2 | 3 | 4 | 5 | 6 | 7 | 8 | 9 |
| 3. Most people believe you are failing in life if you do not feel **Happy** all the time. | 1 | 2 | 3 | 4 | 5 | 6 | 7 | 8 | 9 |
| 4. To get ahead in this world you need to be seen by others as a **Happy** person. | 1 | 2 | 3 | 4 | 5 | 6 | 7 | 8 | 9 |
| 5. I think that society places a great deal of pressure on people to feel **Happy.** | 1 | 2 | 3 | 4 | 5 | 6 | 7 | 8 | 9 |
| 6. Other people generally expect me to feel **Happy**. | 1 | 2 | 3 | 4 | 5 | 6 | 7 | 8 | 9 |
| 7. Others are generally only interested in me when I am feeling **Happy.** | 1 | 2 | 3 | 4 | 5 | 6 | 7 | 8 | 9 |
| 8. I often feel a great deal of pressure from those around me to feel **Happy.** | 1 | 2 | 3 | 4 | 5 | 6 | 7 | 8 | 9 |
| 9. If I am honest, it is really important to me that others see me as someone who is always **Happy.** | 1 | 2 | 3 | 4 | 5 | 6 | 7 | 8 | 9 |

**Supplemental Materials 4: Generic model structure**

In this generic model overview, the outcome (O) refers to either participants’ life satisfaction (LS), the frequency or intensity of their positive (PA) or negative affect (NA), or the intensity of their depression-, anxiety-, or stress-related mood symptoms (i.e., 7 different outcomes). At level 1, the predictor (P) either represents participants’ (group-mean centered) perceived social expectancies to feel happy (SEHS) or not to feel depressed or anxious (SEDAS; i.e., 2 different predictors). At level 2, we always entered nations’ (grand-mean centered) world happiness index (WHI) and evaluated its cross-level interaction with the intercept and person-level predictor.

Level 1:

$$O_{ij}= \beta_{0j}+ \beta_{1j}P_{ij}+\varepsilon_{ij}$$

Level 2:

$$\beta_{0j}=\gamma_{00}+\gamma_{01}\mathrm{WHI}_{j}+\eta_{0j}$$

$$\beta_{1j}=\gamma_{10}+\gamma_{11}\mathrm{WHI}_{j}+\eta_{1j}$$

At Level 1, O*_ij_* represents a subjective well-being (SWB) outcome for participant *i* in country *j*. This outcome is modelled as a function of a random intercept *β_0j_* and a predictor P, for which the effect *β_1j_* is also allowed to vary across countries. Residual scores are represented by ε*_ij_* and are assumed to be normally distributed with ε*_ij_* ~ *N*(0,*δ*). At Level 2, *β_0j_* and *β_1j_* are function of countries’ WHI score. Thus, together, the *γ*s are the fixed or population effects, while the *η*_j_s represent the random effects [with *η_ij_* ~ *N*(0,*ρ*)].

**Supplemental Materials 5: Summary of all multilevel coefficients**

| **Supplemental Table 2.** Summary of multilevel models with SEHS as focal predictor. | | | | | |
| --- | --- | --- | --- | --- | --- |
| Predictor | Estimate | SE | *t* | 95% CI | *p* |
| **Outcome: PA Frequency** | | | | | |
| Intercept | 5.64 | 0.05 | 115.83 | [5.54 ; 5.73] | <.001 |
| SEHS | -0.11 | 0.03 | -3.84 | [-0.16; -0.05] | <.001 |
| WHI | -0.07 | 0.06 | -1.22 | [-0.18; 0.04] | .222 |
| SEHS x WHI | -0.08 | 0.03 | -2.51 | [-0.15; -0.02] | .012 |
| **Outcome: NA Frequency** | | | | | |
| Intercept | 4.83 | 0.08 | 59.39 | [4.67; 4.99] | <.001 |
| SEHS | 0.37 | 0.02 | 15.46 | [0.32; 0.41] | <.001 |
| WHI | 0.14 | 0.10 | 1.47 | [-0.05; 0.33] | .142 |
| SEHS x WHI | 0.07 | 0.03 | 2.61 | [0.02; 0.12] | .009 |
| **Outcome: PA intensity** | | | | | |
| Intercept | 5.67 | 0.05 | 107.26 | [5.57; 5.77] | <.001 |
| SEHS | -0.09 | 0.03 | -3.22 | [-0.14; -0.04] | .001 |
| WHI | -0.07 | 0.06 | -1.20 | [-0.20; 0.05] | .230 |
| SEHS x WHI | -0.08 | 0.03 | -2.40 | [-0.14; -0.01] | .016 |
| **Outcome: NA intensity** | | | | | |
| Intercept | 4.87 | 0.08 | 64.94 | [4.73; 5.02] | <.001 |
| SEHS | 0.37 | 0.03 | 13.65 | [0.31; 0.42] | <.001 |
| WHI | 0.16 | 0.09 | 1.86 | [-0.01; 0.34] | .063 |
| SEHS x WHI | 0.08 | 0.03 | 2.66 | [0.02; 0.14] | .008 |
| **Outcome: Life satisfaction** | | | | | |
| Intercept | 4.37 | 0.05 | 92.88 | [4.28; 4.46] | <.001 |
| SEHS | -0.05 | 0.02 | -2.46 | [-0.10; -0.01] | .014 |
| WHI | 0.14 | 0.06 | 2.56 | [0.03; 0.25] | .010 |
| SEHS x WHI | -0.08 | 0.03 | -2.94 | [-0.13; -0.03] | .003 |
| **Outcome: Depressive symptoms** | | | | | |
| Intercept | 1.78 | 0.04 | 50.25 | [1.71; 1.85] | <.001 |
| SEHS | 0.10 | 0.01 | 10.21 | [0.08; 0.12] | <.001 |
| WHI | 0.02 | 0.04 | 0.38 | [-0.07; 0.10] | .702 |
| SEHS x WHI | 0.04 | 0.01 | 3.16 | [0.01; 0.06] | .002 |
| **Outcome: Anxiety symptoms** | | | | | |
| Intercept | 1.72 | 0.03 | 53.46 | [1.66; 1.79] | <.001 |
| SEHS | 0.09 | 0.01 | 11.70 | [0.08; 0.11] | <.001 |
| WHI | -0.01 | 0.04 | -0.36 | [-0.09; 0.06] | .717 |
| SEHS x WHI | 0.03 | 0.01 | 2.75 | [0.01; 0.04] | .006 |
| **Outcome: Stress symptoms** | | | | | |
| Intercept | 2.06 | 0.03 | 62.69 | [2.00; 2.12] | <.001 |
| SEHS | 0.10 | 0.01 | 12.62 | [0.09; 0.12] | <.001 |
| WHI | 0.06 | 0.04 | 1.44 | [-0.02; 0.13] | .151 |
| SEHS x WHI | 0.03 | 0.01 | 2.91 | [0.01; 0.05] | .004 |

**Note.** Values are graphically summarized in Figure 1 of the main article (panel A). SEHS = Social Expectancies to be Happy Scale; PA = Positive Affect; NA = Negative Affect. WHI = World Happiness Index.

| **Supplemental Table 3.** Summary of multilevel models with SEDAS as focal predictor. | | | | | |
| --- | --- | --- | --- | --- | --- |
| Predictor | Estimate | SE | *t* | 95% CI | *p* |
| **Outcome: PA Frequency** | | | | | |
| Intercept | 5.65 | 0.05 | 115.21 | [5.55; 5.74] | <.001 |
| SEDAS | -0.27 | 0.03 | -9.08 | [-0.33; -0.21] | <.001 |
| WHI | -0.07 | 0.06 | -1.22 | [-0.18; 0.04] | .224 |
| SEDAS x WHI | -0.10 | 0.03 | -2.80 | [-0.17; -0.03] | .005 |
| **Outcome: NA Frequency** | | | | | |
| Intercept | 4.83 | 0.08 | 58.12 | [4.67; 4.99] | <.001 |
| SEDAS | 0.46 | 0.03 | 17.20 | [0.41; 0.51] | <.001 |
| WHI | 0.14 | 0.10 | 1.47 | [-0.05; 0.33] | .142 |
| SEDAS x WHI | 0.04 | 0.03 | 1.45 | [-0.02; 0.10] | .146 |
| **Outcome: PA intensity** | | | | | |
| Intercept | 5.68 | 0.05 | 106.10 | [5.57; 5.78] | <.001 |
| SEDAS | -0.24 | 0.03 | -8.33 | [-0.30; -0.18] | <.001 |
| WHI | -0.07 | 0.06 | -1.19 | [-0.20; 0.05] | .234 |
| SEDAS x WHI | -0.09 | 0.03 | -2.85 | [-0.16; -0.03] | .004 |
| **Outcome: NA intensity** | | | | | |
| Intercept | 4.87 | 0.08 | 63.71 | [4.72; 5.02] | <.001 |
| SEDAS | 0.46 | 0.03 | 16.81 | [0.41; 0.52] | <.001 |
| WHI | 0.17 | 0.09 | 1.86 | [-0.01; 0.34] | .063 |
| SEDAS x WHI | 0.07 | 0.03 | 2.39 | [0.01; 0.14] | .017 |
| **Outcome: Life satisfaction** | | | | | |
| Intercept | 4.37 | 0.05 | 90.68 | [4.28; 4.47] | <.001 |
| SEDAS | -0.23 | 0.02 | -10.45 | [-0.27; -0.19] | <.001 |
| WHI | 0.14 | 0.06 | 2.53 | [0.03; 0.25] | .012 |
| SEDAS x WHI | -0.07 | 0.02 | -2.80 | [-0.12; -0.02] | .005 |
| **Outcome: Depressive symptoms** | | | | | |
| Intercept | 1.78 | 0.04 | 48.96 | [1.71; 1.85] | <.001 |
| SEDAS | 0.14 | 0.01 | 13.56 | [0.12; 0.16] | <.001 |
| WHI | 0.02 | 0.04 | 0.38 | [-0.07; 0.10] | .707 |
| SEDAS x WHI | 0.04 | 0.01 | 3.17 | [0.01; 0.06] | .002 |
| **Outcome: Anxiety symptoms** | | | | | |
| Intercept | 1.73 | 0.03 | 52.27 | [1.66; 1.79] | <.001 |
| SEDAS | 0.11 | 0.01 | 12.20 | [0.09; 0.12] | <.001 |
| WHI | -0.01 | 0.04 | -0.35 | [-0.09; 0.06] | .723 |
| SEDAS x WHI | 0.02 | 0.01 | 1.91 | [-0.01; 0.04] | .057 |
| **Outcome: Stress symptoms** | | | | | |
| Intercept | 2.06 | 0.03 | 61.02 | [1.99; 2.13] | <.001 |
| SEDAS | 0.12 | 0.01 | 13.53 | [0.10; 0.14] | <.001 |
| WHI | 0.06 | 0.04 | 1.42 | [-0.02; 0.13] | .156 |
| SEDAS x WHI | 0.03 | 0.01 | 2.96 | [0.01; 0.05] | .003 |

**Note.** Values are graphically summarized in Figure 1 of the main article (panel B). SEDAS = Social Expectancies not to be Depressed or Anxious Scale; PA = Positive Affect; NA = Negative Affect. WHI = World Happiness Index.

**Supplemental Materials 6: Statistical alternatives to evaluate robustness of found effects**

Due to the cross-sectional nature of our study, our interpretations are limited to a correlational level (i.e., investigating a symmetrical relation). In contrast, linear regression analyses require researchers to arbitrarily specify an outcome and predictor (e.g., social expectancies to be happy predicting various well-being outcomes), which introduces an *asymmetry* in their relation. In a multilevel context, this arbitrary decision may occasionally yield different conclusions (60).

To rule out the possibility that our results were affected by this arbitrary decision, and to by-pass the role of the inherent asymmetry in multilevel modeling, we performed two other series of analyses. First, we simply switched outcome and predictor in all multilevel models, meaning that we now used the various well-being indicators to predict individual differences in either (a) the social pressure to be happy or (b) not to be sad. Akin to the models presented in the main text, we each time group-mean centered the predictor of interest, and grand-mean centered countries’ WHI score. Next, in a second approach, we group-mean standardized both the outcome and predictor to overcome the observed asymmetry in multilevel modeling (61). In this approach, the fixed effect represents the average observed correlation between social expectancies and well-being in our cross-national sample. Adding countries’ grand-mean standardized WHI score at the national level indicates how this relation then varies as a function of between-country differences in WHI. Note that the intercept following this approach is always zero, and is therefore not estimated (i.e., both standardized values have a mean score of zero). Consequently, also the intercept’s interaction with countries’ WHI score cannot be estimated.

The results for both approaches can be found in Supplementary Table 4 (SEHS) and 5 (SEDAS). Comparing the significance (with α = .05) of the fixed effects and their associated cross-level interaction learns that our results largely replicate across the different analytic approaches (deviations are bolded). Consequently, these robustness analyses suggest that our conclusions are not driven by a particular statistical strategy.

| **Supplemental Table 4.** Summary of different analytic strategies for SEHS models. | | | | | | |
| --- | --- | --- | --- | --- | --- | --- |
| Model | SEHS as predictor | | SEHS as outcome | | SEHS & well-being standardized | |
|  | Estimate | *p* | Estimate | *p* | Estimate | *p* |
| **PA Frequency** | | | | | | |
| Intercept | 5.64 | <.001 | 6.01 | <.001 |  |  |
| VAR | -0.11 | <.001 | -0.09 | <.001 | -0.10 | <.001 |
| WHI | -0.07 | .222 | 0.12 | .143 |  |  |
| VAR x WHI | -0.08 | .012 | -0.06 | .018 | -0.06 | .016 |
| **NA Frequency** | | | | | | |
| Intercept | 4.83 | <.001 | 6.01 | <.001 |  |  |
| VAR | 0.37 | <.001 | 0.19 | <.001 | 0.27 | <.001 |
| WHI | 0.14 | .142 | 0.12 | .144 |  |  |
| VAR x WHI | 0.07 | .009 | 0.03 | .027 | 0.04 | .017 |
| **PA intensity** | | | | | | |
| Intercept | 5.67 | <.001 | 6.01 | <.001 |  |  |
| VAR | -0.09 | .001 | -0.06 | .001 | -0.07 | .001 |
| WHI | -0.07 | .230 | 0.12 | .151 |  |  |
| VAR x WHI | -0.08 | .016 | -0.05 | .019 | -0.05 | .018 |
| **NA intensity** | | | | | | |
| Intercept | 4.87 | <.001 | 6.01 | <.001 |  |  |
| VAR | 0.37 | <.001 | 0.16 | <.001 | 0.24 | <.001 |
| WHI | 0.16 | .063 | 0.12 | .149 |  |  |
| VAR x WHI | 0.08 | .008 | 0.03 | .020 | 0.04 | .016 |
| **Life satisfaction** | | | | | | |
| Intercept | 4.37 | <.001 | 6.01 | <.001 |  |  |
| VAR | -0.05 | .014 | -0.04 | **.068** | -0.05 | .0322 |
| WHI | 0.14 | .010 | 0.12 | **.155** |  |  |
| VAR x WHI | -0.08 | .003 | -0.09 | .003 | -0.07 | .002 |
| **Depressive symptoms** | | | | | | |
| Intercept | 1.78 | <.001 | 6.01 | <.001 |  |  |
| VAR | 0.10 | <.001 | 0.44 | <.001 | 0.21 | <.001 |
| WHI | 0.02 | .702 | 0.12 | .153 |  |  |
| VAR x WHI | 0.04 | .002 | 0.15 | .012 | 0.06 | .004 |
| **Anxiety symptoms** | | | | | | |
| Intercept | 1.72 | <.001 | 6.01 | <.001 |  |  |
| VAR | 0.09 | <.001 | 0.50 | <.001 | 0.22 | <.001 |
| WHI | -0.01 | .717 | 0.12 | .152 |  |  |
| VAR x WHI | 0.03 | .006 | 0.11 | .043 | 0.04 | .015 |
| **Stress symptoms** | | | | | | |
| Intercept | 2.06 | <.001 | 6.01 | <.001 |  |  |
| VAR | 0.10 | <.001 | 0.56 | <.001 | 0.24 | <.001 |
| WHI | 0.06 | .151 | 0.12 | .152 |  |  |
| VAR x WHI | 0.03 | .004 | 0.10 | **.074** | 0.05 | .015 |

**Note.** Deviations in significance from main analyses are bolded (with α = .05). SEHS = Social Expectancies to be Happy Scale; PA = Positive Affect; NA = Negative Affect. WHI = World Happiness Index. VAR = Variable that either refers to SEHS or focal well-being variable.

| **Supplemental Table 5.** Summary of different analytic strategies for SEDAS models. | | | | | | |
| --- | --- | --- | --- | --- | --- | --- |
| Model | SEDAS as predictor | | SEDAS as outcome | | SEDAS & well-being standardized | |
|  | Estimate | *p* | Estimate | *p* | Estimate | *p* |
| **PA Frequency** | | | | | | |
| Intercept | 5.65 | <.001 | 5.62 | <.001 |  |  |
| VAR | -0.27 | <.001 | -0.18 | <.001 | -0.22 | <.001 |
| WHI | -0.07 | .224 | 0.12 | .111 |  |  |
| VAR x WHI | -0.10 | .005 | -0.07 | <.001 | -0.07 | .001 |
| **NA Frequency** | | | | | | |
| Intercept | 4.83 | <.001 | 5.62 | <.001 |  |  |
| VAR | 0.46 | <.001 | 0.20 | <.001 | 0.30 | <.001 |
| WHI | 0.14 | .142 | 0.12 | .110 |  |  |
| VAR x WHI | 0.04 | .146 | 0.04 | **.007** | 0.04 | **.025** |
| **PA intensity** | | | | | | |
| Intercept | 5.68 | <.001 | 5.62 | <.001 |  |  |
| VAR | -0.24 | <.001 | -0.13 | <.001 | -0.18 | <.001 |
| WHI | -0.07 | .234 | 0.12 | .117 |  |  |
| VAR x WHI | -0.09 | .004 | -0.05 | <.001 | -0.06 | <.001 |
| **NA intensity** | | | | | | |
| Intercept | 4.87 | <.001 | 5.62 | <.001 |  |  |
| VAR | 0.46 | <.001 | 0.17 | <.001 | 0.28 | <.001 |
| WHI | 0.17 | .063 | 0.12 | .114 |  |  |
| VAR x WHI | 0.07 | .008 | 0.04 | <.001 | 0.05 | .002 |
| **Life satisfaction** | | | | | | |
| Intercept | 4.37 | <.001 | 5.62 | <.001 |  |  |
| VAR | -0.23 | <.001 | -0.19 | <.001 | -0.20 | <.001 |
| WHI | 0.14 | .012 | 0.12 | **.112** |  |  |
| VAR x WHI | -0.07 | .005 | -0.05 | .005 | -0.06 | .003 |
| **Depressive symptoms** | | | | | | |
| Intercept | 1.78 | <.001 | 5.62 | <.001 |  |  |
| VAR | 0.14 | <.001 | 0.52 | <.001 | 0.26 | <.001 |
| WHI | 0.02 | .707 | 0.12 | .1118 |  |  |
| VAR x WHI | 0.04 | .002 | 0.14 | .004 | 0.06 | .002 |
| **Anxiety symptoms** | | | | | | |
| Intercept | 1.73 | <.001 | 5.62 | <.001 |  |  |
| VAR | 0.11 | <.001 | 0.47 | <.001 | 0.22 | <.001 |
| WHI | -0.01 | .723 | 0.12 | .112 |  |  |
| VAR x WHI | 0.02 | .057 | 0.09 | .060 | 0.04 | **.046** |
| **Stress symptoms** | | | | | | |
| Intercept | 2.06 | <.001 | 5.62 | <.001 |  |  |
| VAR | 0.12 | <.001 | 0.54 | <.001 | 0.25 | <.001 |
| WHI | 0.06 | .156 | 0.12 | .112 |  |  |
| VAR x WHI | 0.03 | .003 | 0.14 | .003 | 0.06 | .002 |

**Note.** Deviations in significance from main analyses are bolded (with α = .05). SEDAS = Social Expectancies not to be Depressed or Anxious Scale; PA = Positive Affect; NA = Negative Affect. WHI = World Happiness Index. VAR = Variable that either refers to SEDAS or focal well-being variable.
